# Disappearance of a thrombotic microangiopathy-like glomerular lesion in a patient with a placental site trophoblastic tumor after hysterectomy 

**DOI:** 10.5414/CNCS109440

**Published:** 2018-09-20

**Authors:** Masato Sawamura, Atsushi Komatsuda, Mizuho Nara, Masaru Togashi, Hideki Wakui, Naoto Takahashi

**Affiliations:** 1 Department of Hematology, Nephrology, and Rheumatology, Akita University Graduate School of Medicine, and; 2 Department of Life Science, Akita University Graduate School of Engineering Science, Akita, Japan

**Keywords:** nephrotic syndrome, placental site trophoblastic tumor, TMA-like lesion

## Abstract

A 32-year-old woman presented with amenorrhea after a normal childbirth and subsequently developed nephrotic syndrome. Renal biopsy showed a thrombotic microangiopathy (TMA)-like glomerular lesion with deposits of immunoglobulins, complements, and fibrinogen. Increased serum levels of the beta subunit of human chorionic gonadotropin, abnormal uterine findings from imaging studies, and endometrial biopsy findings suggested gestational trophoblastic disease. She was diagnosed with a placental site trophoblastic tumor (PSTT) after hysterectomy and, following treatment, her proteinuria disappeared. Follow-up renal biopsy showed the disappearance of the TMA-like lesion. To our knowledge, this is the first case report of the pathological remission of renal disease associated with PSTT.

## Introduction 

Gestational trophoblastic diseases (GTD) are a rare group of pregnancy-related tumors. Malignant GTD include choriocarcinoma, placental site trophoblastic tumors (PSTT), and epithelioid trophoblastic tumors [1]. PSTT is a relatively newly recognized disease entity. Since its initial description in 1976, more than 500 cases have been reported [2]. 

The association of PSTT with renal diseases is extremely rare, and only 6 cases have been reported in the English literature [3, 4, 5, 6, 7]. In these cases, 3 patients developed thrombotic microangiopathy (TMA)-like glomerular lesions with clinical remission after hysterectomy [3, 4, 6]. Here, we present the case of a TMA-like glomerular lesion in a patient with nephrotic syndrome and PSTT. A novel finding in our case is that the disappearance of the TMA-like lesion was documented based on renal biopsy in addition to clinical remission. Another novel finding is the presence of anti-cardiolipin antibody. We also review the clinicopathological features in previously reported cases of nephrotic syndrome associated with PSTT. 

## Case report

**Figure 1.**
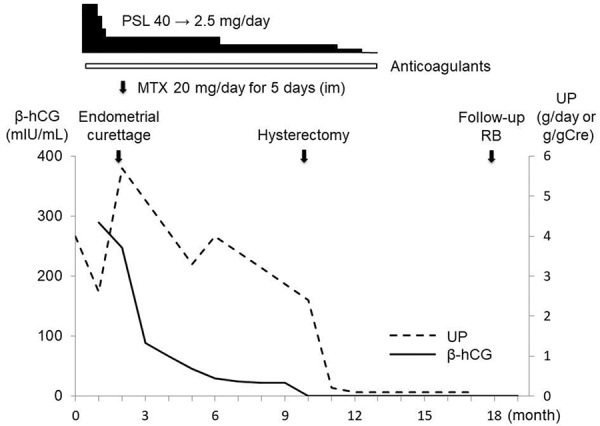
Clinical course. β-hCG = human β subunit of chorionic gonadotropin; Cre = creatinine; im = intramuscular injection; MTX = methotrexate; PSL = prednisolone; RB = renal biopsy; UP = urinary protein.

A 32-year-old woman was admitted to our hospital with progressive edema. She had a history of photosensitivity, malar rash, and oral ulcers from childhood. She presented with amenorrhea 6 months after normal childbirth. A home pregnancy test was positive and she visited a local hospital 2 months prior to her admission. She was diagnosed with a miscarriage by sonography that showed no fetal sac. She developed progressive edema from 1 month (weight gain of 4 kg) prior to her admission. The clinical course after admission is shown in Figure 1. 

On admission, her body temperature was 36.9 °C, blood pressure 138/89 mmHg, and the pulse rate 99 beats/minutes with a regular rhythm. A physical examination showed no malar rash or oral ulcers. There were moderate edemas on the face and both legs. No crackles were audible in the chest, and the heart sounds were normal. The liver and spleen were not palpable. 

Her erythrocyte count, hemoglobin level, leukocyte count (lymphocytes 6.2%), and platelet count were 479 × 10^4^/µL, 14.2 g/dL, 14,000/µL, and 18.2 × 10^4^/µL, respectively. Urinalysis showed proteinuria with mild hematuria. Total urinary protein level for 24 hours was 4.0 g. Her serum total protein was 4.5 g/dL, albumin 2.1 g/dL, blood urea nitrogen 16.1 mg/dL, creatinine 0.46 mg/dL, lactate dehydrogenase 245 U/L, haptoglobin (2-1 type) 172 mg/dL, C3 94 mg/dL, C4 11 mg/dL, and CH50 44 U/mL. Tests for antinuclear and anti-dsDNA antibodies were negative, but the concentration of anti-cardiolipin IgG antibody was 23.4 U/mL (normal < 10 U/mL). Serological tests for rheumatoid factor, human immunodeficiency virus, hepatitis B virus, and hepatitis C virus were negative. 

Computed tomography (CT) revealed bilateral pleural effusion, ascites, and swelling of the uterus with low-density areas. 

**Figure 2.**
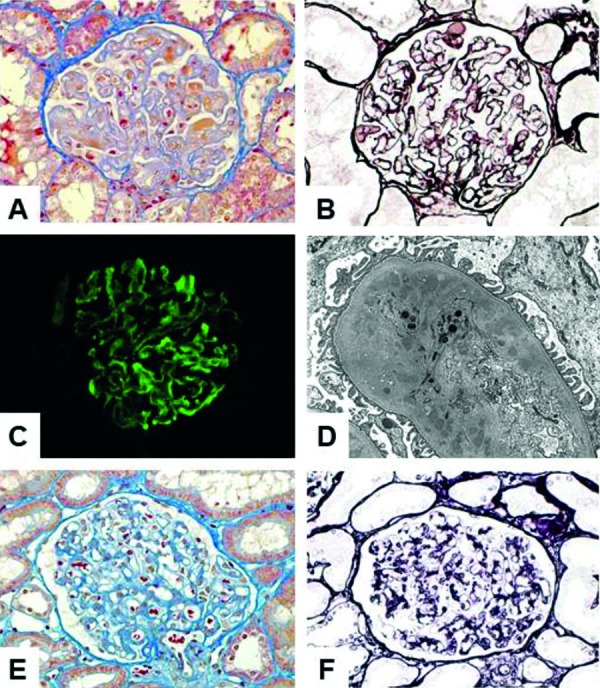
A, B: Light microscopy of initial renal biopsy specimens shows thickening of the glomerular capillary walls, thrombus-like structures within the glomerular capillary lumina, and a double-contour appearance along the glomerular capillary walls (A: Masson’s trichrome stain × 400; B: Periodic acid-methenamine-silver stain × 400). C: Immunofluorescence microscopy shows positive staining for IgM along the glomerular capillary walls. D: Electron microscopy shows the expansion of the glomerular subendothelial space with electron-dense aggregates. E, F: Light microscopy of follow-up renal biopsy specimens shows normal glomerular structures (E: Masson’s trichrome stain × 400; F: Periodic acid-methenamine-silver stain × 400).

Due to the presence of nephrotic syndrome, a renal biopsy was performed. Light microscopy showed thickened glomerular capillary walls with lumina occluded by thrombus-like structures and a double-contour appearance along the glomerular capillary walls (Figure 2A, B). There was mild mesangial expansion and proliferation. Tubulointerstitial structures were preserved, and there were no apparent vascular changes. Immunofluorescence microscopy showed 2+ staining for IgM, IgA, and fibrinogen, 1+ staining for C1q, and 0.5+ staining for IgG, κ, λ, and C3 along the glomerular capillary walls (Figure 2C). Electron microscopy showed large aggregates in the glomerular capillary lumina as well as expansion of the glomerular subendothelial space with amorphous electron-dense aggregates, but no deposits in the mesangial area. There were no alterations of the podocyte foot processes (Figure 2D). 

Considering her previous history that consisted of lymphopenia, renal disorder, and positive anti-cardiolipin IgG antibody, an initial diagnosis of systemic lupus erythematosus was made according to the 2012 SLICC criteria [8]. Since renal biopsy showed a thrombotic microangiopathy (TMA)-like glomerular lesion, antiphospholipid syndrome (APS) was suspected. She was initially treated with prednisolone, beraprost sodium, and warfarin. Although the progressive edema decreased, proteinuria (~ 2 – 3 g/day) persisted. 

Based on the abnormal CT findings of the uterus, her serum human β-subunit of chorionic gonadotropin (β-hCG) level was measured. The results showed that the level had increased to 289.2 mIU/mL (normal < 0.5 mIU/mL). Endometrial cytology showed proliferation of intermediate trophoblasts with abnormal cell nuclei. Endometrial curettage was performed, and methotrexate therapy was initiated. Since the pathological diagnosis was an exaggerated placental site and her serum β-hCG level had decreased to 87.9 mIU/mL, she was followed up at our outpatient clinic 1 month after chemotherapy. 

However, follow-up imaging studies revealed abnormal uterine findings suggestive of a trophoblastic tumor. Total hysterectomy was performed 9 months after her first admission. A pathological examination showed that the tumor was primarily composed of a population of intermediate trophoblastic cells. The tumor cells that had invaded the myometrium and vessels were strongly positive for human placental lactogen, but weakly positive for hCG. Thus, she was diagnosed with PSTT. Two months after the hysterectomy, her proteinuria disappeared and her serum β-hCG level normalized. 

Eight months after the hysterectomy, a follow-up renal biopsy was performed. Light microscopy revealed the disappearance of the TMA-like glomerular lesion (Figure 2E, F). Furthermore, immunofluorescence studies showed negative staining for immunoglobulins, complements, and fibrinogen. 

## Discussion 

Our patient had nephrotic syndrome complicated with PSTT. Renal biopsy showed a TMA-like glomerular lesion. Following the removal of PSTT, the proteinuria disappeared. Therefore, nephrotic syndrome due to a TMA-like lesion was considered to be caused by PSTT. The pathological improvement of the TMA-like lesion was confirmed by follow-up biopsy. 

The association of renal diseases with PSTT is extremely rare. Table 1 summarizes clinicopathological features of PSTT from 6 previously reported cases [3, 4, 5, 6, 7], as well as our case. The age of the patients ranged between 21 and 42 years. Most patients presented with nephrotic-range proteinuria, hypoalbuminemia, and normal renal function. Renal pathological findings were TMA-like glomerular lesions in 5 cases [3, 4, 6], membranous nephropathy in 1 [5], and lupus nephritis (possibly a variant) in 1 [7]. In cases of TMA-like glomerular lesions, occlusive deposits or thrombus-like structures within the glomerular capillary lumina have been reported, similar to the present case. In these cases, glomerular deposits of immunoglobulins, complements, and fibrinogen are commonly observed. By electron microscopy, large aggregates in the glomerular capillary lumina and amorphous electron-dense aggregates within the subendothelial space were observed in 1 case [6], which is similar to the present case. However, vascular changes that are characteristic of the classical form of TMA were not described in these cases. Hysterectomy with or without chemotherapy was performed for all patients with PSTT-associated renal diseases. Except for 1 patient who died of sepsis [4], the disappearance of proteinuria after successful therapy suggests a strong correlation between renal diseases and PSTT. The accumulation of similar cases is required for further studies. 

The pathogenesis of TMA-like glomerular lesions associated with PSTT was not elucidated in the previously reported cases [3, 4, 6]. Common immunohistochemical findings described above support a role for immune complexes and/or intravascular coagulation. Young et al. [4] suggested that abundant IgM glomerular deposits could reflect the presence of antibodies bound to specific antigens in the tumor and that IgM antibodies may be trapped in deposits resulted from intravascular coagulation. Unknown circulating factors supplied by the tumor cells may stimulate the development of complement-dependent TMA-like glomerular lesions, which have been described in most reported cases of renal diseases associated with PSTT. 

Glomerular lesions observed in our patient with anti-cardiolipin IgG antibodies are similar to those in patients with APS nephropathy [9]. Antiphospholipid antibodies may have direct roles in the development of TMA and ASP vasculopathy. However, our patient did not meet the criteria for APS [9] and did not show APS vasculopathy. 

In conclusion, a review of the literature revealed that a TMA-like glomerular lesion may occur in patients with PSTT and may resolve after successful surgical treatment. To ensure correct diagnosis and therapy for GTD-associated renal diseases, renal biopsies need to be considered in women who develop renal symptoms following spontaneous abortion or normal childbirth. 

## Funding 

None. 

## Conflict of interest 

The authors declare no conflict of interest. 

**Table 1.** Clinicopathological features in previously reported cases of PSTT-associated renal diseases and in our case.

Author (reference, year)	Age (yr)	UP	S-Alb (g/dL)	S-Cr (mg/dL)	Max β-HCG (mIU/mL)	Findings of glomerular lesion	Glomerular deposits	Therapy	Outcome
Eckstein et al. 1982 [3]	21	5 g/day	2.4	0.68	362	TMA-like lesion	Immunoglobulins, F	Chemo → Hyst	Remission
Young et al. 1985 [4]	30	4+	2.1	ND	413	TMA-like lesion	M, G, κ, λ, F	Hyst → Chemo	Died
Young et al. 1985 [4]	33	4+	ND	ND	10,000	TMA-like lesion	M, A, F	Hyst	Remission
Batra et al. 2007 [5]	28	2.8 g/day	2.2	0.8	210	MN	M, A, G, C3	Hyst	Remission
Mazzucco et al. 2011 [6]	42	12.3 g/day	2.1	72*	1,685	TMA-like lesion	M, κ, λ, C4, C1q, F	Hyst → Chemo	Remission
Xiao et al. 2014 [7]	31	> 7 g/day	2.6	0.57	95.4	LN variant?	M, A, G, C3, C1q	Hyst	Remission
Present case	32	4.0 g/day	2.1	0.46	289.2	TMA-like lesion	M, A, G, κ, λ, C3, C1q, F	Chemo → Hyst	Remission

A = IgA; Alb = albumin; Chemo = chemotherapy; Cr = creatinine; F = fibrinogen; G = IgG; HCG = human chorionic gonadotropin; Hyst = hysterectomy; LN = lupus nephritis; M = IgM; MN = membranous nephropathy; ND = not described; PSTT = placental site trophoblastic tumor; S = serum; TMA = thrombotic microangiopathy; UP = urinary protein; yr = year. *Cr clearance (mL/min).
